# Unilateral Pulmonary Agenesis about a Case Revealed by an Acute Respiratory Infection in a Young Infant

**DOI:** 10.1155/2023/8110952

**Published:** 2023-03-09

**Authors:** S. Aimée Kissou, Souleymane B. W. Adjaba, Jacqueline Tamini, Hélène Traore

**Affiliations:** ^1^Department of Pediatrics, CHU Sourô Sanou (CHUSS), BP 676, Bobo-Dioulasso, Burkina Faso; ^2^Higher Institute of Health Sciences (INSSA), Nazi BONI University (UNB), Bobo-Dioulasso, Burkina Faso

## Abstract

Pulmonary agenesis is a rare congenital anomaly of unknown cause, consisting of a complete absence of the lung parenchyma, bronchi, and vascularization. It may or not be associated with other malformations. The authors report a case of right pulmonary agenesis diagnosed in a four-month-old infant who had no previous pathological history and was growing well. The circumstance of discovery was respiratory distress during an infectious episode. The diagnosis was suspected on a standard chest X-ray and confirmed on a chest CT scan. There are no other associated defects. The evolution was good after antibiotic therapy. While bilateral pulmonary agenesis is incompatible with life, the unilateral form may remain unrecognized until adulthood. The prognosis depends mainly on the importance of the associated malformations.

## 1. Background

Pulmonary agenesis is a rare congenital malformation, consisting of a complete absence of the lung parenchyma, bronchi, and vascularization [1]. Although some risk factors are sometimes mentioned, the etiology of this malformation remains unknown. Its prevalence worldwide is estimated at 34 cases per million live births [2]. In half of the cases, it is associated with other malformations, notably cardiovascular, gastrointestinal, genitourinary, and musculoskeletal, which worsen the prognosis in unilateral forms. These forms are usually latent, unlike the bilateral form which is incompatible with life. The authors report a case of isolated right pulmonary agenesis discovered during a respiratory infection in a 4-month-old infant.

## 2. Case Presentation

GS, a four-month-old female infant, was referred for respiratory distress complicating symptomatology that began two days earlier, consisting of rhinorrhea, cough, and moderate fever. She was born to a 27-year-old woman, 3^rd^ gest and 3^rd^ pare, at the end of an unremarkable pregnancy. Toxoplasmosis, syphilis, rubella, cytomegalovirus, and herpes serologies had not been performed. There was no notion of parental consanguinity, no particular family defect, and no exposure to drugs or other teratogenic agents during the pregnancy. A single obstetrical ultrasound was performed at 34 weeks of amenorrhea, with no mention of any abnormality. GS was born in a health center. It was a full term (39 weeks + 4 days) and vaginal delivery, with a birth weight of 3300 grams and a good adaptation to extra-uterine life. The Apgar score was, respectively, 8/10 and 10/10 at the 1^st^ and the 5^th^ minutes of life. No notion of dyspnea or other pathological history of the patient was reported by the parents. On admission, the infant's general condition was preserved, consciousness was clear, and palmoplantar staining was normal. The Body temperature was 37.4°C. Peripheral oxygen saturation (SpO2) was 99% on room air. Respiratory rate was 46 cycles/min and pulse 127 beats/min. The chest was symmetrical with tachypnea, immobility of the right hemithorax, and moderate signs of respiratory struggle in the form of intercostal pulling and nasal wing flapping. The vesicular murmur was prominent in the left hemithorax and weak in the right hemithorax. Heart sounds were deviated to the right, with regular tachycardia without murmurs or overtones. The rest of the physical examination was unremarkable. In addition, the infant was in good nutritional condition, with a weight of 6700 g and a height of 65 cm, so, a weight/height Z-score below the median and a height/age Z-score above the median.

**Figure 1:**
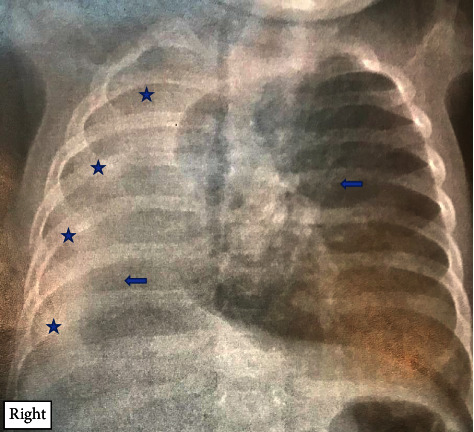
Frontal chest radiograph in a 4-month-old infant, showing right thoracic opacity (stars), hypertrophy and mediastinal herniation of the left lung (arrows), and lack of visualization of the left stem bronchus.

The diagnosis of right pneumonia was considered. A frontal chest radiograph showed a right white lung with herniation of mediastinal contents in the right hemithorax (Figure 1).

**Figure 2:**
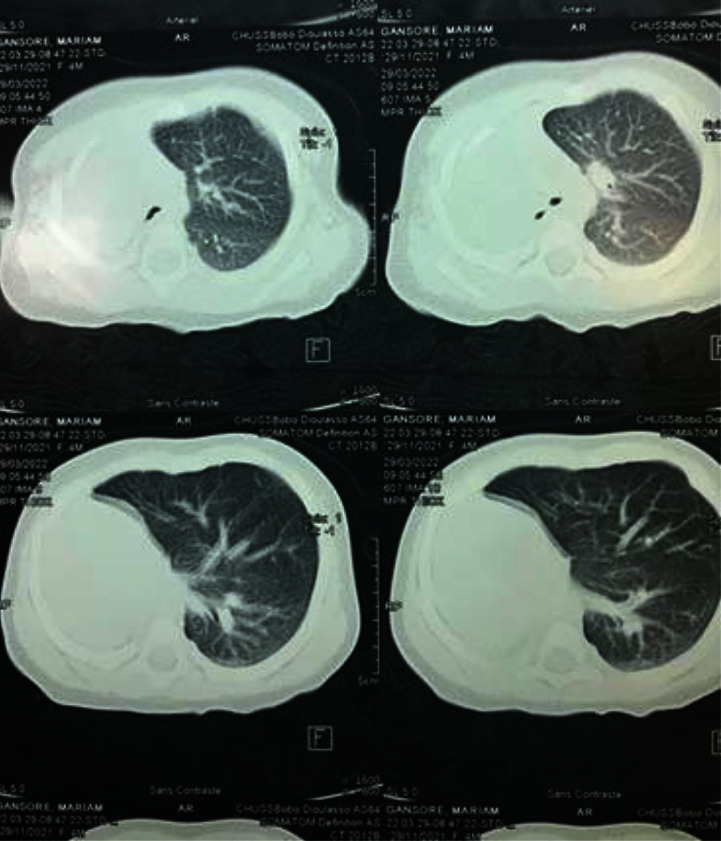
Chest CT scan in a 4-month-old infant showing agenesis of the right lung parenchyma, absence of a right stem bronchus, compensatory hypertrophy of the left lung, which extended to the right hemithorax, causing deviation of the mediastinum and responsible for dextrocardia, and a single left pulmonary artery.

A thoracoabdominal CT scan confirmed the diagnosis of pulmonary aplasia by showing the absence of parenchyma, stem bronchus, and vascularization (Figure 2).

The complementary radiological workup (cardiac ultrasound and abdominal CT scan) did not reveal any associated malformation. The evolution was favorable with a rapid improvement of the respiratory distress with antibiotic treatment associated with hygienic and dietary measures (regular washing of the nose, fractioning of meals). The infant was released after 8 days of hospitalization. She was seen again two weeks later and showed no functional signs of disease.

## 3. Discussion

Lung agenesis is a rare congenital anomaly, which may or may not be associated with other malformations. Its incidence is estimated to be between 1/10,000 and 1/15,000 based on autopsy series [3]. Although its etiology remains unknown, some believe that its pathogenesis is related to genetic (consanguinity), infectious (rubella virus), or nutritional (vitamin A or folic acid deficiency) factors [3]. Schneider's classification (1912) modified by Boyden in 1955 distinguishes the following three types of congenital anomalies of lung development [3, 4]:

- Type 1 which corresponds to agenesis of one lung, with complete absence of the lung, the stem bronchus, and the vascularization
- Type 2 which corresponds to pulmonary aplasia in which a rudimentary blind stem bronchus is found, without any pulmonary parenchyma
- Type 3 which is hypoplasia with a rudimentary bronchial tree in which most of the bronchi are blind at the end; vascularization and pulmonary parenchyma sometimes bronchiectasis are present

While bilateral pulmonary agenesis is incompatible with life, the prognosis of unilateral forms is often related to the presence or absence of other associated malformations. These malformations may involve the respiratory system, heart, skeleton, digestive system, or urinary tract [5–10]. In the absence of associated malformations, unilateral pulmonary agenesis may remain unrecognized and sometimes asymptomatic and diagnosed late in infancy or even in adulthood [3, 10, 11]. However, manifestations in neonates or infants can be observed, sometimes after an infectious episode of the single lung, frequently causing respiratory distress as in the patient reported here [12–16]. In our patient, antibiotic therapy was instituted despite the absence of evidence of a bacterial infection, for fear of a possible superinfection and taking into account the single lung context.

Although the predominance of left lung agenesis compared to right lung agenesis has been reported by some authors, the different series reported do not confirm this trend [4, 17]. In addition, right-sided pulmonary agenesis would have a more severe prognosis because of a greater displacement of the mediastinum leading to compression of the trachea by the aortic arch and limitation of its growth by the superior vena cava. In this form, the apparent dextrocardia is normal and related to the mediastinal deviation to the right.

The diagnosis of pulmonary agenesis can be made before or after birth. In children or adults, the standard radiograph usually shows an opaque hemithorax, distension of the contralateral lung, part of which is often herniated on the other side of the midline with more or less severe pinching of the homolateral intercostal spaces [1]. However, thoracic computed tomography with an injection of contrast medium remains the reference examination to make the diagnosis, allowing visualization of the agenesis of the pulmonary artery [1, 5].

Other more invasive imaging techniques such as bronchoscopy and ventilation-perfusion scintigraphy are not available in our context and are not essential for the diagnosis of pulmonary agenesis.

In the absence of radical curative treatment of pulmonary agenesis, management will consist of follow-up with prevention of infections to preserve the single lung as much as possible. General hygiene measures vaccinations, especially against pneumococcus, *Haemophilus influenzae b,* and influenza should be administered. Early treatment of any infection should be ensured by the education of the family.

## 4. Conclusion

Unilateral pulmonary agenesis is a rare congenital malformation whose prognosis depends not only on the type of malformation but also on other associated anomalies. It may reveal itself early with spontaneous dyspnea or during an infection of the unique lung, or remain asymptomatic for a long time. The diagnosis can be made by a thoracic CT scan. Therapeutic management, which is not yet consensual, is generally based on medical treatment in symptomatic patients and the prevention of infections.

## Data Availability

No data were available.
